# Association of strong opioids and antibiotics prescribing with GP burnout: a retrospective cross-sectional study

**DOI:** 10.3399/BJGP.2022.0394

**Published:** 2023-05-16

**Authors:** Alexander Hodkinson, Salwa S Zghebi, Evangelos Kontopantelis, Christos Grigoroglou, Darren M Ashcroft, Mark Hann, Carolyn A Chew-Graham, Rupert A Payne, Paul Little, Simon de Lusignan, Anli Zhou, Aneez Esmail, Maria Panagioti

**Affiliations:** NIHR School for Primary Care Research and NIHR Greater Manchester Patient Safety Translational Research Centre, University of Manchester, Manchester.; NIHR School for Primary Care Research, University of Manchester, Manchester.; NIHR School for Primary Care Research; Division of Informatics, University of Manchester, Manchester.; NIHR School for Primary Care Research; Manchester Centre for Health Economics, University of Manchester, Manchester.; NIHR School for Primary Care Research; NIHR Greater Manchester Patient Safety Translational Research Centre; Centre for Pharmacoepidemiology and Drug Safety, University of Manchester, Manchester.; NIHR School for Primary Care Research, University of Manchester, Manchester.; School of Medicine, Keele University, Keele.; Exeter Collaboration for Academic Primary Care, University of Exeter Medical School, Exeter.; Primary Care Research Centre, University of Southampton, Southampton.; Nuffield Department of Primary Care Health Sciences, University of Oxford, Oxford; Royal College of General Practitioners Research and Surveillance Centre, London.; NIHR School for Primary Care Research, University of Manchester, Manchester.; NIHR School for Primary Care Research, University of Manchester, Manchester.; NIHR School for Primary Care Research and NIHR Greater Manchester Patient Safety Translational Research Centre, University of Manchester, Manchester.

**Keywords:** antibiotics, burnout, primary care, hazardous prescribing, opioids, patient safety

## Abstract

**Background:**

Prescribing of strong opioids and antibiotics impacts patient safety, yet little is known about the effects GP wellness has on overprescribing of both medications in primary care.

**Aim:**

To examine associations between strong opioid and antibiotic prescribing and practice- weighted GP burnout and wellness.

**Design and setting:**

A retrospective cross-sectional study was undertaken using prescription data on strong opioids and antibiotics from the Oxford- Royal College of General Practitioners Research and Surveillance Centre linking to a GP wellbeing survey overlaying the same 4-month period from December 2019 to April 2020.

**Method:**

Patients prescribed strong opioids and antibiotics were the outcomes of interest.

**Results:**

Data for 40 227 patients (13 483 strong opioids and 26 744 antibiotics) were linked to 57 practices and 351 GPs. Greater strong opioid prescribing was associated with increased emotional exhaustion (incidence risk ratio [IRR] 1.19, 95% confidence interval [CI] = 1.10 to 1.24), depersonalisation (IRR 1.10, 95% CI = 1.01 to 1.16), job dissatisfaction (IRR 1.25, 95% CI = 1.19 to 1.32), diagnostic uncertainty (IRR 1.12, 95% CI = 1.08 to 1.19), and turnover intention (IRR 1.32, 95% CI = 1.27 to 1.37) in GPs. Greater antibiotic prescribing was associated with increased emotional exhaustion (IRR 1.19, 95% CI = 1.05 to 1.37), depersonalisation (IRR 1.24, 95% CI = 1.08 to 1.49), job dissatisfaction (IRR 1.11, 95% CI = 1.04 to 1.19), sickness–presenteeism (IRR 1.18, 95% CI = 1.11 to 1.25), and turnover intention (IRR 1.38, 95% CI = 1.31 to 1.45) in GPs. Increased strong opioid and antibiotic prescribing was also found in GPs working longer hours (IRR 3.95, 95% CI = 3.39 to 4.61; IRR 5.02, 95% CI = 4.07 to 6.19, respectively) and in practices in the north of England (1.96, 95% CI = 1.61 to 2.33; 1.56, 95% CI = 1.12 to 3.70, respectively).

**Conclusion:**

This study found higher rates of prescribing of strong opioids and antibiotics in practices with GPs with more burnout symptoms, greater job dissatisfaction, and turnover intentions; working longer hours; and in practices in the north of England serving more deprived populations.

## INTRODUCTION

Opioids are commonly used for the treatment of pain and include medicines such as morphine, fentanyl, and tramadol. In England, prescribing of opioids between 2008 and 2018 increased by 34%, with >231 million prescriptions dispensed in primary care in 2018–2019 alone.^1^ Non- medical use, prolonged use, misuse, and use without medical supervision can lead to opioid dependence, other serious health problems, and death.^2^ Worldwide, in 2017, an estimated 53.4 million people were on opioids, with opioids making up two-thirds of deaths related to drug misuse.^3^

Antibiotic resistance is also a major challenge to health care. The era of modern medicine has depended on the effective control of communicable diseases, of which many are bacterial in their origin.^4^ Faced with a situation where novel antibiotic agents are in short supply, the need to conserve the existing ‘supply’ of antibiotics becomes ever clearer. Antimicrobial stewardship encompasses a wide range of processes and interventions that are designed to ensure that antibiotics are used in the most effective manner.^5^^,^^6^ However, Public Health England’s National Infection Service recently found that as many as 23% of all antibiotic prescriptions in general practices may have been inappropriate.^7^ Optimising opioid and antibiotic prescribing are highly important policy targets globally.

Many medication optimisation strategies focus on identifying and resolving potentially inappropriate prescribing, such as through pharmacist medication reviews and the use of information technology (for example, PINCER).^8^^,^^9^ However, practice characteristics and staff wellness factors might be equally as important in reducing overprescribing and preventing patient safety incidents as patient factors.^10^ There is increasing evidence internationally that the wellness of physicians including GPs is associated with poor quality of care outcomes including medication and prescription errors.^11^ Furthermore, a study among 232 practising GPs suggested that changes at both the practice and individual level would help to promote a healthier work environment for staff and patients, and improve patient safety generally.^12^ However, this evidence has been criticised because it is mostly based on self-reported quality of care and patient safety data by doctors.^13^ Moreover, this self-reported evidence has not focused on prescribing specifically.

**Table:** How this fits in

Prescribing has important implications for patient safety; this is particularly the case for high-risk medications such as strong opioids, and medications where there may be public health implications such as antibiotics. Physician wellness such as burnout can also have a significant impact on the productivity of healthcare organisations, intentions to leave medical practice, and both the quality and safety of patient care. At present, it is unclear if there is an association between the wellness of GPs within general practices and overprescribing of strong opioids and antibiotics in primary care in England. Over a 4-month period this study found higher prescribing of strong opioids and antibiotics among GPs with burnout symptoms, job dissatisfaction, and turnover intentions; working longer hours; and in practices based in the north of England serving more deprived populations.

A key marker of healthcare staff wellness is burnout, which is defined as a work- related syndrome involving three key dimensions: emotional exhaustion; depersonalisation; and personal accomplishment.^14^

Closely related characteristics that associate with burnout include turnover intention (intention to leave job within 5 years) and job satisfaction.^15^ Importantly, physician wellness has been increasingly seen as an organisational quality indicator. Furthermore, GP wellness has been viewed as an organisational problem, and thus wellness measures could be analysed as a practice-rather than an individual-level characteristic of GPs.

The aim of this study was to assess the association of the volume and potentially hazardous prescribing of strong opioids and antibiotics as the outcome of interest with key characteristics of general practices (with a focus on GP burnout) as the key exposure. Prescription data were obtained from the UK Oxford-Royal College of General Practitioners (RCGP) Research and Surveillance Centre (RSC) from December 2019 to April 2020, and the exposure burnout/wellbeing variables were surveyed across the same time period.

## METHOD

### Data source

A retrospective cross-sectional study was conducted involving prescription outcome data that was linked with GP wellbeing responses (exposure) from an online survey from December 2019 to April 2020 using the RSC. The prescription data and survey responses both covered the same 4-month period to ensure consistency when linking the two datasets in the cross- sectional study.

The RSC is an internationally renowned source of information holding pseudo- anonymised individual-level GP primary care data.^16^^,^^17^ It provides patient- level data including prescription records and information about diagnosis, which have been carefully curated into variables historically using the Read terminology and more recently the Systematised Nomenclature of Medicine (SNOMED) Clinical Terms (CT).^18^^,^^19^ The RSC sentinel data include the monitoring of upper and lower respiratory tract infections (URTIs and LRTIs, respectively), and the careful differentiation of new and ongoing episodes of care.^20^ RSC data also capture every prescription issued in primary care and have previously been used to conduct research on antibiotic use.^21^

The authors of the current study provided the RSC with coded product and medical concepts using similar strategies to those used in their earlier work involving opioids^22^ and antibiotics.^23^ A list of the Read codes, which constitute the current study’s inclusion criteria for products and medical conditions, are provided in Supplementary Table S1.

The GP survey involved 10 items and was intended to reach 350–400 GPs across 70 different practices. The distribution of the survey was done using random sampling in house and was sent online to participating GP practices through the RCGP RSC using the Survey Monkey platform. Each participating GP received a £20 payment to their GP practice. A copy of the full survey is provided in Supplementary Information S1.

Reporting in the study was undertaken in accordance with the Strengthening the Reporting of Observational Studies in Epidemiology (STROBE) guidelines.^24^

### Study populations

The study included patients aged ≥18 years in the 4-month period with:

- any indication of chronic pain (including post-operative pain);
- prescribed strong opioids; and
- any respiratory tract infection and prescribed antibiotics.

The full medical and product code list is provided in Supplementary Table S1.

### Covariates

#### Survey and GP wellness scores. 

GP characteristics collected in the survey included practice identification code (including NHS region), age, sex, full- time equivalence (FTE), and seven key outcomes associated with GP wellness including emotional exhaustion (EE) and depersonalisation (DP) subscales of burnout,^25^ sickness-presenteeism,^26^ work– life balance,^27^ diagnostic uncertainty,^28^ job satisfaction,^29^ and intention to leave their job within 5 years (turnover intention).

The single-item measures for GP burnout with the highest factor loading on EE (‘feelings of being burnt out’) and DP (‘feelings of becoming callous towards people’) were used as the primary measures of burnout. Both items were initially measured on the ordinal scale of 1 (low) to 7 (high), and the other wellness factors were also measured on an ordinal scale.^25^

#### RSC record-linked primary care data

GP surveillance data include all prescriptions issued including the dosage, which were converted to count data based on the total number of tablets per patient. As the data were delivered in various ways (that is, tablets, capsules, ampoule, and patches), for consistency in this study each prescription was standardised to the single measure of mgs to allow for adjustment for potency in the modelling. Demographic patient data included baseline characteristics of age, sex, ethnicity (that is, White, Asian, Black, mixed, or other), consultation type (that is, clinical administration, electronic consultation, face to face, telephone, home visit, and unspecified), related comorbidities (that is, immunocompromised, asthma, and chronic respiratory disease), mental health symptoms/episodes (that is, anxiety, depression, mental health referral, obsessive–compulsive disorder [OCD], panic attack, post-traumatic stress disorder, and stress), smoking (that is, active, ex-smoker, non-smoker, or non-specific), drinking habit (alcoholic, hazardous, safe, and non-drinker), history of other available medication use (that is, hypnotic, antidepressant, and anxiolytic), and socioeconomic status measured using the English Index of Multiple Deprivation (IMD) 2015 quintiles.^30^

The IMD is an aggregate measure of relative deprivation across seven domains (income, employment, education and skills, health and disability, crime, barriers to housing and services, and living environment) using an area-based model at a low geography (average of 1500 people) and via practice/patient postcodes. Overall IMD is calculated as a weighted mean across the seven domains, with income and employment deprivation given the largest weight (22.5%), followed by health and disability and education and skills deprivation (13.5%), and the other three domains are given equal weights (9.3%).^31^

### Statistical analysis

As some of the GP wellness scores were missing from the original survey, missing data were imputed using the R package ‘MICE: Multivariate Imputation by Chained Equations’.^32^ As it was not possible to directly link the individual GP wellness response data from the survey to the main RSC prescription data without attaining consent from the GPs, in this study the data were linked at the practice level. This meant a practice-weighted score for GP wellness was calculated for each practice. The practice-weighted scores were calculated for all of the nine items in the survey (GP age, FTE, EE, DP, sickness-presenteeism, work–life balance, diagnostic uncertainty, job satisfaction, and turnover intention) using the average weight function *svyby* in the *survey* package of R.^33^ The variable FTE was used as a predictor to improve approximation of the practice-weighted scores. The GP wellness variable was then included in the multilevel model described below.

Descriptive statistics described the characteristics of the patient population involved, and spearman rank correlations assessed associations across all the practice-weighted GP wellness scores.

The patient-level association of volume of prescribing (response variable) with the practice-weighted wellness scores (EE, DP, job satisfaction, sickness-presenteeism, diagnostic uncertainty, turnover intention, and work–life balance), practice (NHS region, average GP age, and FTE), and patient factors (sex, age, ethnicity, IMD, consultation type, comorbidities, symptoms/episodes, and history of other medication use related to condition) were examined by fitting a multilevel generalised linear model (GLM) with a negative binomial distribution for each medication independently.

A negative binomial model is favoured here because the prescription data were found to be overdispersed (that is, the magnitude of the variance exceeds the magnitude of the mean). The Poisson regression model often underestimates the standard errors with the presence of overdispersion.^34^ Thus, empirically, a negative binomial model gives more accurate estimates than Poisson regression in most cases.^35^^,^^36^ Data availability drove the decision as to which variables were to be included in the models. In both models a random effects intercept of practice ID was introduced, and the incidence risk ratio (IRR) and 95% confidence interval (CI) estimates were used to determine the association with increased prescribing. All *P*-values were two-sided, and variables with *P*-values <0.05 were regarded as significant in the model.^37^ Variance inflation factors (VIFs) were examined for multicollinearity, with scores <5 considered as moderately correlated and scores ≥5 considered highly correlated.^38^ If a VIF was violated, a sensitivity analysis removing highly correlated variables from the model was applied. All analysis was done in R (version 4.0.5), and the *MASS* package was used to fit the GLMs.^39^

## RESULTS

The cross-sectional study of just over 4 months included 67 practices, of which 57 (85%) (involving 351 GPs) could be linked to the RSC primary care data for 40 227 patients who met the study inclusion criteria. The 10 (15%) practices excluded from the study involved GP registrars consulting at multiple practices at once and therefore they could not be consigned to one unique practice ID.

### Descriptive patient and service characteristics

The median response rate of the GPs across the practices was 39% (range 12%–91%). In total, 13 483 (34%) users of strong opioids and 26 744 (66%) users of antibiotics were identified. The median age of strong opioid users was 65 (range 52–77) years, and for antibiotics it was 50 (24–70) years (Tables 1 and 2).

**Table 1.** Descriptive characteristics of the patients who were strong opioid users

**Strong opioid prescribing characteristics in patients with chronic pain**	***n* (%)^a^^,^^b^**
**Total patients**	13 483

**Age, years, median (range)**	65 (52–77)

**Sex**	
Male	4938 (37)
Female	8363 (62)

**Ethnicity**	
Asian	171 (1)
Black	59 (0.4)
Mixed	46 (0.3)
Other	42 (0.3)
White	10 171 (75)

**District**	
City/town	9021 (67)
Conurbation	2275 (17)
Rural	1281 (10)

**IMD patient quintiles**	
1	2259 (17)
2	2215 (16)
3	2697 (20)
4	2753 (20)
5	2646 (20)

**Alcohol use**	
Alcoholism	850 (6)
Hazardous	3848 (29)
Non-drinker	3538 (26)
Safe	4033 (30)

**Anxiety episode count^c^**	
None	13 255 (98)
>1	46 (0.3)

**Depression episode count^d^**	
None	13 019 (97)
>1	282 (2)

**Mental health referral^e^**	
None	13 183 (98)
>1	118 (1)

**Stress episode counts^f^**	
None	13 295 (99)
>1	6 (0.04)

**>1 hypnotic prescription**	801 (6)

**NHS region**	
London	45 (0.3)
Midlands and east	1588 (12)
North	7246 (54)
South	4604 (34)

**Clinical system**	
EMIS web TPP	5759 (43)
SystmOne	7724 (57)

a *Unless otherwise stated.*

b *Most values do not sum to 100% because their remainder were missing data or not coded.*

c *Number of Anxiety counts: 0 = 13 255; 1 = 39; 2 = 5; 3 = 1; 4 = 1.*

d *Number of depression episode counts: 0 = 13 019; 1 = 193; 2 = 47; 3 = 22; 4 = 13; 5 = 2; 6 = 2; 7 = 2; 10 = 1.*

e *Mental health referral count: 0 = 13 183; 1 = 93; 2 = 22; 3 = 1; 4 = 2.*

f *Stress count: 0 = 13 295; 1 = 5; 4 = 1. IMD = Index of Multiple Deprivation.*

**Table 2.** Descriptive characteristics of the patients who were antibiotic users

**Antibiotic prescribing characteristics in patients with RTIs**	***n* (%)^a^^,^^b^**
**Total patients**	26 744

**Age, years, median (range)**	50 (24–70)

**Sex**	
Male	11 028 (41)
Female	15 289 (57)

**Ethnicity**	
Asian	794 (3)
Black	218 (1)
Mixed	208 (1)
Other	164 (1)
White	18 788 (70)

**District**	
City/town	17 752 (66)
Conurbation	4753 (18)
Rural	2671 (10)

**IMD patient quintiles**	
1	3970 (15)
2	3979 (15)
3	5165 (19)
4	5308 (20)
5	6743 (25)

**Asthma**	
No	21 266 (80)
Yes	5051 (19)

**Chronic respiratory disease**	
No	23 094 (86)
Yes	3223 (12)

**Smoking status**	
Active smoker	4158 (16)
Ex-smoker	10 512 (39)
Non-smoker	6917 (26)
Non-specific	74 (0.3)

**>1 antibiotic prescribed**	16 477 (62)

**NHS region**	
London	249 (1)
Midlands and east	2920 (11)
North	14 466 (54)
South	9109 (34)

**Clinical system**	
EMIS web TPP	14 569 (54)
SystmOne	12 175 (46)

a *Unless otherwise stated.*

b *Most values do not sum to 100% because their remainder were missing data or not coded. IMD = Index of Multiple Deprivation. RTI = respiratory tract infection.*

At least 62% and 57% of users of strong opioids and antibiotics, respectively, were female. Over 70% of the patients in both treatment groups were of White ethnicity, over 66% were based in a city/town, and 54% involved patients registered with an NHS practice in northern England. IMD quintile scores were classified >2 in over 60% of patients in both medication groups (Tables 1 and 2).

Nearly 30% of strong opioid users were classified as ‘hazardous’ alcohol drinkers, and 39% of antibiotic users were ex-smokers (Tables 1 and 2). At least 19% and 12% of antibiotic users had asthma and chronic respiratory disease, respectively (Table 2).

### Characteristics of the practice-weighted GP wellness scores

The median number of GP responders per practice was 5 (interquartile range [IQR] 4). On average GPs reported EE a few times a week (median 3.3, IQR 1.5), experiences of DP once a week (median 3.7, IQR 1.3), sickness- presenteeism >5 times a year (median 3.3, IQR 0.5), find at least 11%–15% of patients difficult to diagnose (median 3.0, IQR 0.6), and were dissatisfied with their current work–life balance (median 3.2, IQR 1.2). On average GPs also reported a moderate likelihood of leaving direct patient care within 5 years (median 2.1, IQR 0.9) and were generally dissatisfied with their job (median 2.5, IQR 0.9) (data not shown).

**Figure 1.**
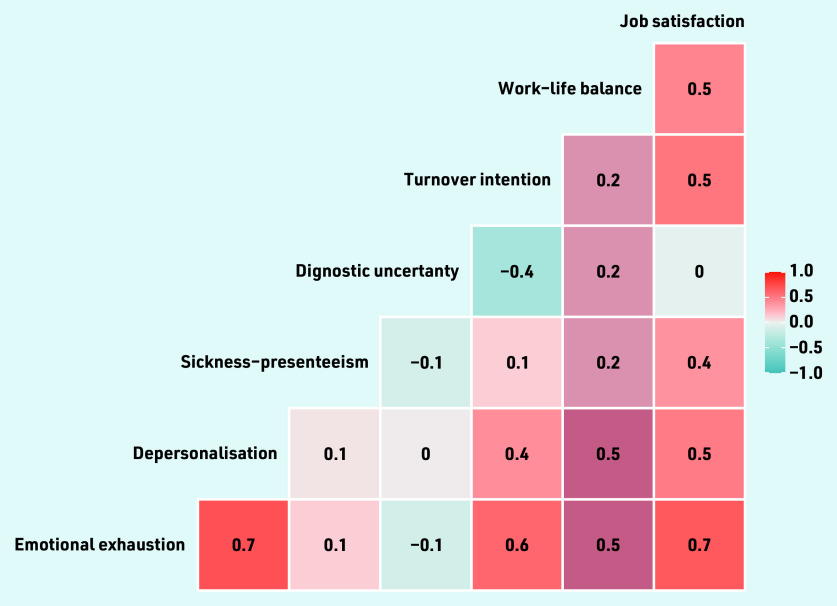
*Correlation plot of the 57 practice-weighted GP wellness scores.*

Figure 1 provides the correlations of the practice-weighted GP burnout scores (EE and DP) against the other practice- weighted GP wellness factors. EE was strongly associated with increased DP (ρ = 0.7), job dissatisfaction (ρ = 0.7) and turnover intention (ρ = 0.6). The correlations between the other factors were low to medium (ρ = 0 to 0.5).

### Association of increased prescribing of strong opioids and antibiotics with GP wellness and other patient/practice factors

#### Strong opioids

Based on 13 483 (34%) users of strong opioids, increased prescribing was significantly associated with a practice-weighted higher risk of EE (IRR 1.19, 95% CI = 1.10 to 1.24), DP (IRR 1.10, 95% CI = 1.01 to 1.16), job dissatisfaction (IRR 1.25, 95% CI = 1.19 to 1.32), diagnostic uncertainty (IRR 1.12, 95% CI = 1.08 to 1.19), and turnover intention (IRR 1.32, 95% CI = 1.27 to 1.37) in GPs. Increased opioid prescribing was also found in practices with longer working hours (FTE) (IRR 3.95, 95% CI = 3.39 to 4.61) and in practices in the north of England (IRR 1.96, 95% CI = 1.61 to 2.33) compared with practices in the south (as the north of England is the reference group, IRR has been reversed; see Supplementary Table S2 for full results).

In terms of patient factors, strong opioid prescribing was significantly associated with male patients (IRR 1.18, 95% CI = 1.15 to 1.22), being older (IRR 1.02, 95% CI = 1.01 to 1.02), more deprived (IMD quintile 5, IRR 1.51, 95% CI = 1.22 to 1.89), and with alcoholism (IRR 1.13, 95% CI = 1.08 to 1.18) or ‘hazardous’ drinking status (IRR 1.09, 95% CI = 1.06 to 1.13). Reduced prescribing of strong opioids was found in Black (IRR 0.45, 95% CI = 0.24 to 0.82) and mixed (IRR 0.45, 95% CI = 0.21 to 0.97) ethnicity patients compared with White patients, and in patients with a higher number of depression episodes (IRR 0.94, 95% CI = 0.91 to 0.96), mental health referrals (IRR 0.79, 95% CI = 0.70 to 0.88), and OCD episodes (IRR 0.12, 95% CI = 0.02 to 0.64). The VIF scores were all <5, therefore no variables were removed.

#### Antibiotics

Based on 26 744 (66%) users of antibiotics, increased prescribing was significantly associated with a higher practice-weighted risk of EE (IRR 1.19, 95% CI = 1.05 to 1.37), DP (IRR 1.24, 95% CI = 1.08 to 1.49), job dissatisfaction (IRR 1.11, 95% CI = 1.04 to 1.19), sickness- presenteeism (IRR 1.18, 95% CI = 1.11 to 1.25), and turnover intention (IRR 1.38, 95% CI = 1.31 to 1.45) in GPs. Increased antibiotics prescribing was also found in practices with longer working hours (IRR 5.02, 95% CI = 4.07 to 6.19) and in practices in the north of England (IRR 1.56, 95% CI = 1.12 to 3.70) compared with practices in the south of England (as the north of England is the reference group, IRR has been reversed; see Supplementary Table S3).

With regards to patient factors, increased antibiotic prescribing was significantly associated with male patients (IRR 1.15, 95% CI = 1.11 to 1.19), being older (IRR 1.01, 95% CI = 1.01 to 1.02), and with higher deprivation (IMD quintile 5, IRR 1.16, 95% CI = 1.08 to 1.25). There was a significant reduction in antibiotic prescribing in Black patients (IRR 0.72, 95% CI = 0.62 to 0.82), those diagnosed with asthma (IRR 0.86, 95% CI = 0.82 to 0.90) or chronic respiratory disease (IRR 0.79, 95% CI = 0.75 to 0.83), and who were active (IRR 0.65, 95% CI = 0.62 to 0.69) or ex-smokers (IRR 0.79, 95% CI = 0.75 to 0.82). No multicollinearity was present in the model (see Supplementary Table S3).

## DISCUSSION

### Summary

In this large national cross-sectional study involving 57 practices comprising 351 GPs with 40 227 patients, it was found that prescribing of strong opioids over 4 months in 13 483 patients was greatest in GPs working in practices in the north of England, who worked longer hours, and who showed increased levels of practice-weighted burnout (EE and DP), job dissatisfaction, diagnostic uncertainty, and turnover intention. For antibiotic use in 26 744 patients over 4 months, it was found that there was increased prescribing in practices in the north of England, in GPs working longer hours, and those with increased levels of practice-weighted burnout (EE and DP), job dissatisfaction, sickness-presenteeism, and turnover intention.

### Strengths and limitations

To the authors’ knowledge, this is the first study with use of a novel approach to link GP survey responses weighted at practice- level to GP patient surveillance records to investigate the relationship between the prescribing of strong opioids and antibiotics and GP wellness across practices in England.

The study has several limitations. First, it was not an experimental study design, meaning unmeasurable confounding for prescribing of both drugs is possible.

Second, by not being able to directly link the GP survey responses to the surveillance health records without the GPs consent meant it was necessary to calculate the practice-weighted scores for GP wellness factors. This in turn affected the ability to directly assess for potential clustering factors of the GPs with their prescribing characteristics. Furthermore, the accuracy for estimating the practice-weighted scores may have been impeded by the low response rate, which on average was 39% across practices. This may have led to overestimation or even an underestimation of the average practice burnout/wellbeing scores. However, this is still higher than the 12% response rate attained in the UK’s Tenth National GP work-life Survey in 2019.^40^ One consideration was to try to account for this low response rate in the study design by using imputation methods.^41^ However, there are significant complexities surrounding the best ways to impute the missing GP responses and how reliable this would be, given there was such limited demographic information about the GPs and practices themselves from the survey. Bayesian models using missing at random and missing not at random algorithms have been proven effective when imputing missing response data,^42^ but need to be properly tested in this environment.

Third, the decision not to apply a form of univariable regressions to observe how each covariate altered the treatment response and establish an order of importance for each of the GP wellbeing factors may have weakened the modelling. However, given the importance of each wellbeing factor the authors opted to include them all in the final model. In terms of the patient- level factors such as patient demographic characteristics and complications/symptoms, these variables were chosen based on input from the clinicians involved in the study. Practice- weighted wellness scores (EE, DP, job satisfaction, sickness-presenteeism, diagnostic uncertainty, turnover intention, and work– life balance) were selected based on existing frameworks that have studied the relationship between occupational distress in physicians and poor quality of patient care outcomes.^43^^,^^44^

Fourth, the study overlapped with the start of the COVID-19 pandemic, meaning some patients may have been subject to more relaxed medicine management, low morale, and predominantly remote care,^45^ which will have had some impact on antibiotic prescribing.^46^ This was not adjusted for in the analysis.

Fifth, the number of practices recruited in this study was based on available funding for the questionnaire collection, rather than a formal sample size calculation. However, the patient sample was not small, and the study did find statistically significant associations between the key variables of interest (GP wellbeing and overprescribing), and overprescribing of antibiotics and opioids. However, the authors of the current study strongly encourage larger studies to further investigate these associations, especially in a prospective research design.

Sixth, as detailed in the Method, dosage data were provided in different forms of delivery. Thus, the authors had to standardise the data to the unique measure based on mgs. However, as it was not possible to standardise up to 13% of the prescription data to mgs, these data therefore had to be removed from the cohort. Undertaking a sensitivity analysis was considered to adjust for the loss of these data, but because of the uncertainty on the dose provided to the patient the authors decided against this.

Finally, as a result of the relatively low number of general practices (*n* = 57), it was not possible to assess disparities between rural versus inner city/urban areas, which is important to understand from a UK policy perspective.

### Comparisons with existing literature

The current findings are consistent with the fast-growing research evidence that shows that physician burnout may risk the quality of care provided to patients.^43^ To date, however, most of this evidence has been based on patient safety outcomes, self- reported by physicians. The current findings add to this body of evidence, demonstrating that GP burnout is associated with objectively reported overprescribing of strong opioids and antibiotics, by utilising novel linkages between a GP survey and patient data contained in a large health database.

A previous study has shown that primary care providers who overprescribed opioids to treat patients with chronic pain often exhibit signs of burnout and feel unable to help patients overcome their complex challenges.^47^ However, that study involved a very small sample of only 19 primary care clinicians and eight nurses, and they used a qualitative ethnographic approach that limited any quantification of the association between prescribing of opioids and burnout.

Furthermore, while inappropriate antibiotic use has been linked to the emergence of drug resistance, which contributes directly to increased medical costs,^48^ the impact of antibiotic overprescribing due to worsening GP wellness has not been formally assessed. One such effort^49^ had tried to assess the association between physician wellness (burnout and empathy) and antibiotic prescribing for RTIs in 36 primary care practices in Northeast Ohio, US. They found no association between physician wellness and antibiotic prescribing, but these findings might be more reflective of a lack of statistical power in their study sample.

### Implications for research and practice

The findings from the current practice- level approach to burnout and quality of patient care have important policy implications. Policies are urgently needed to mitigate burnout in UK general practice, commissioned as practice-embedded workforce wellness programmes rather than external support services made available to individual members of the workforce (for example, GPs) who may experience burnout. Such practice-embedded workforce wellness programmes could produce further improvements to the mainstream category of medication safety improvement strategies, which focus mostly on identifying patients ‘at risk’ rather than workforce or general practices at risk.

Monitoring and understanding healthcare worker wellness requires conducting health- related surveys and surveillance, but the combining of these data with prescription (surveillance) electronic health records is more challenging as it requires consent to attribute the GPs who are responsible for prescribing the medication.^50^^,^^51^ Obtaining such consent is considered a controversial area for many physicians, and the authors are not aware of any such novel and successful efforts to date. If a large enough response rate can be achieved, then the association of wellness factors and prescription characteristics can be assessed with high reliability considering missing responses. The authors encourage similar innovative efforts to investigate this as a possible model in future research designs.

## Data

This study used pseudonymised patient- level data from the Oxford-Royal College of General Practitioner (RCGP) Research and Surveillance Centre (RSC). These data can be accessed for ethically approved research by applying via: https://orchid.phc.ox.ac.uk
